# Stimulation of Natural Killer T Cells by Glycolipids

**DOI:** 10.3390/molecules181215662

**Published:** 2013-12-16

**Authors:** Brian L. Anderson, Luc Teyton, Albert Bendelac, Paul B. Savage

**Affiliations:** 1 Department of Chemistry and Biochemistry, Brigham Young University, Provo, UT 84602, USA; 2 Department of Immunology, The Scripps Research Institute, La Jolla, CA 92602, USA; 3 Committee on Immunology and Department of Pathology, Howard Hughes Medical Institute, University of Chicago, Chicago, IL 60637, USA

**Keywords:** glycolipid, natural killer T cell, cytokine, innate immunity

## Abstract

Natural killer T (NKT) cells are a subset of T cells that recognize glycolipid antigens presented by the CD1d protein. The initial discovery of immunostimulatory glycolipids from a marine sponge and the T cells that respond to the compounds has led to extensive research by chemists and immunologists to understand how glycolipids are recognized, possible responses by NKT cells, and the structural features of glycolipids necessary for stimulatory activity. The presence of this cell type in humans and most mammals suggests that it plays critical roles in antigen recognition and the interface between innate and adaptive immunity. Both endogenous and exogenous natural antigens for NKT cells have been identified, and it is likely that glycolipid antigens remain to be discovered. Multiple series of structurally varied glycolipids have been synthesized and tested for stimulatory activity. The structural features of glycolipids necessary for NKT cell stimulation are moderately well understood, and designed compounds have proven to be much more potent antigens than their natural counterparts. Nevertheless, control over NKT cell responses by designed glycolipids has not been optimized, and further research will be required to fully reveal the therapeutic potential of this cell type.

## 1. Introduction

Natural killer T (NKT) cells are a subset of T cells that recognize a variety of lipid and glycolipid antigens. The most studied subpopulation of NKT cells, invariant NKT cells (iNKT or Type 1 NKT cells), have a T cell receptor (TCR) with an variable (V) *α*14 and joining (J) *α*18 gene chain rearrangement in mice and a homologous V*α*24-J*α*18 rearrangement in humans [1,2,3]. In addition, the TCR has a restricted selection of *β* TCR chains [4]. In contrast to T-helper cells and cytotoxic T cells, the TCR of iNKT cells recognizes antigens that are presented by the non-classical MHC-like membrane-bound cell-surface glycoprotein CD1d [5,6].

CD1d, mainly expressed on B-cells, dendritic cells, macrophages, and epithelial cells, presents lipid-containing molecules to the TCR of iNKT cells [5]. The structure of CD1d consists of two chains: a heavy chain comprised of three extracellular domains (*α*1*−α*3) and a *β*2 microglobulin chain [5,7]. Cd1d is one of five isoforms found in humans (CD1a-e) with a homologous CD1d isoform in mice [8,9]. It is hypothesized that these isoforms exist so they can recycle through multiple intracellular compartments where they sample lipid antigens that are present and/or are trafficked to different endocytic compartments [10]. For example, CD1a traffics through early and recycling endosomes on its way to the cell surface [11], whereas CD1b and CD1d are localized in the late endosome and lysosome where microbial lipids accumulate during infections [12,13,14].

Unlike its major histocompatibility complexes (MHC) counterparts, CD1d presents lipids, not peptides, to the TCR of iNKT cells. The groove on CD1d is narrower, deeper, and more hydrophobic than MHC class-I and II binding grooves. The antigen-binding groove forms two hydrophobic pockets, termed the A' and F' pockets. These pockets generally accommodate and bind long carbon lipid tails from a variety of lipid-containing molecules [9,12]. Presented antigenic lipids or glycolipids interact with the TCR of iNKT cells and this interaction subsequently activates iNKT cells, releasing a variety of cytokines and chemokines that modulate and/or stimulate the immune system [2,15]. 

After stimulation from a CD1d-bound antigen, iNKT cells produce, within hours, large amounts of cytokines [16]. Released cytokines can promote two distinct immune responses. One group of cytokines (e.g., interleukin (IL)-2, interferon(IFN)-γ, and tumor necrosis factor-α) leads to a proinflammatory T helper 1 (TH1) response. TH1 responses are employed to combat and control bacterial, viral, and parasitic infections [2,17]. Cytokines, such as IL-4, IL-5, IL-6, IL-10, and IL-13, promote an immunoregulatory T helper 2 (TH2) response. Many autoimmune diseases such as type I diabetes, multiple sclerosis, lupus, and rheumatoid arthritis can be ameliorated through TH2-mediated responses [17,18,19].

iNKT cells modulate innate and adaptive immune responses. Released cytokines can activate adaptive cells, such as T and B cells, and innate cells, such as dendritic cells and NK cells [20,21]. These bidirectional signals can be received through cell surface receptors, such as the TCR recognizing lipid-bound CD1, costimulatory receptors (CD40, CD70, OX40), or cytokines. For example, activation of iNKT cells results in rapid maturation of dendritic cells and B cells [12,17]. Because of their immunological role, governing the stimulation of iNKT cells is a therapeutically relevant goal.

### 1.1. The Model iNKT Antigen: α-GalCer

**Figure 1:**
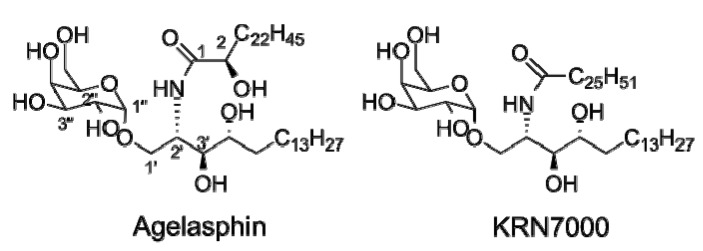
Representative structures of an agelasphin and KRN7000.

In 1993, Kirin Pharmaceuticals, a pharmaceutical subsidiary of Kirin Brewery Inc., reported results from a systematic screening of marine natural products for anti-tumor activity. Their efforts led to the isolation of a family of glycolipids termed “agelasphins” (a sample agelasphin is presented in Figure 1). Agelasphins consist of saccharides that are α*-* or β*-*linked to a phytosphingosine-containing ceramide backbone. The different agelasphins vary mostly by the composition and length of their lipid tails, as well as the saccharide composition. Their initial screenings demonstrated that α*-*linked galactose- containing agelasphin glycolipids were significantly more potent against B16 mouse melanoma cells than the β*-*linked agelasphins [22,23,24].

The promising anti-tumor activities of agelasphins, led Morita *et al.* [25] to perform a structure-activity relationship (SAR) study with the intention of finding a potent commercially viable anti-tumor agent. Their efforts led to the synthesis of KRN7000, more commonly referred to as α*-*galactosylceramide (α*-*GalCer). This SAR study also established precedence for the importance of the C3' hydroxyl on anti-tumor activity, the causality of longer ceramide acyl chains towards better anti-tumor activity, and the optimal phytosphingosine chain length (18 carbon phytosphingosine scaffold) [26].

The importance of α*-*GalCer was realized in 1997, when α*-*GalCer was shown to be a CD1d-restricted iNKT cell antigen [27]. Consequently, this analog became the model and primary antigen in the study of iNKT cell stimulation [9]. Over the next several years, the immunoregulatory role of iNKT cells, and the subsequent importance of α*-*GalCer, became more apparent. Many well-documented studies have focused on elucidating the diseases that are affected by iNKT cells [2]. These studies have generally done one of three things to survey iNKT cell involvement in murine and human diseases: (1) compared the iNKT cell numbers between control and diseased individuals; (2) monitored the effect of CD1d or iNKT cell depletion on the disease; or (3) administered α*-*GalCer to see its effect on the disease in question [28]. In this way, iNKT cells have been implicated in microbial infections and multiple autoimmune diseases (e.g., type 1 diabetes, multiple sclerosis, rheumatic arthritis, asthma) [1].

### 1.2. The Continuing Search for iNKT Cell Antigens

Although α*-*GalCer is the standard model iNKT cell antigen, it has at least two limitations that inhibit its therapeutic effectiveness. First, after iNKT cell stimulation by CD1d-bound α*-*GalCer, the immune system releases both TH1 and TH2 cytokines that, in some cases, can counteract one another in modulating the immune system. This was suggested by Kronenberg and coworkers [29] with the observation that activated iNKT cells quickly release the immunostimulatory IFN-γ cytokine and the immunomodulating cytokine IL-4. Second, α*-*GalCer can stimulate iNKT cells too potently causing a cytokine storm; that is, iNKT cells release a massive amount of cytokines leading to iNKT cell anergy or inactivation of iNKT cells [12]. This iNKT cell anergy was shown by Uldrich *et al.* [30] and Parekh *et al.* [31]. In both studies iNKT cells exhibited a hyporesponsiveness to subsequent α*-*GalCer challenges after administration of α*-*GalCer. Because of the therapeutic potential of iNKT cells and the limitations of α*-*GalCer, an effort to find more effective iNKT cell antigens has ensued.

It has been widely accepted that glycolipids from marine sponges (*i.e.*, agelasphins) are not natural antigens for iNKT cells. Consequently, substantial effort has been expended in identifying exogenous and endogenous antigens for NKT cells, and series of discoveries have revealed the breadth of iNKT cell antigens. Exogenous antigens have been identified in multiple human pathogens including bacteria, fungi, and parasites. The presence of endogenous iNKT cell antigens is implicated in the maturation of these cells and their autoreactivity, and multiple candidate antigens have been reported. In addition to natural antigens for iNKT cells, synthetic variants have been designed and prepared in efforts to modify the immunostimulatory and immunomodulatory properties of iNKT. In this review, the structures of exogenous and endogenous natural antigens will be presented. In addition, designed antigens for iNKT cells will be described.

## 2. Exogenous Antigens for iNKT Cells

### 2.1. Sphingomonas Glycosphingolipids

**Figure 2:**
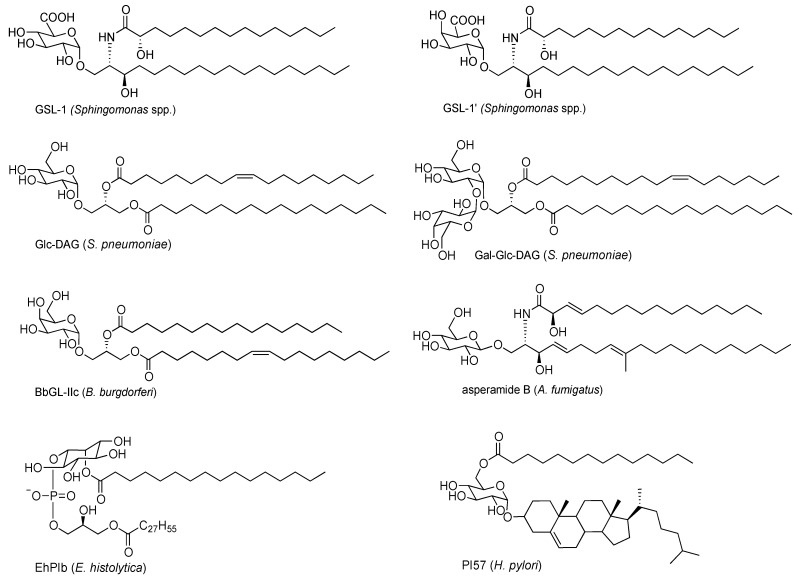
iNKT cell antigens derived from pathogenic sources.

The *Sphingomonadaceae* family of bacteria substitutes glycosylceramides in their outer membranes in place of the lipopolysaccharides found in most Gram-negative bacteria. Various groups have shown that heat-killed *Sphingomonadaceae* spp. bacteria stimulate iNKT cells [32,33,34]. Further characterization of bacterial extracts led to the discovery of glycosphingolipid-1 (GSL-1) and GLS-1' (Figure 2) as antigens for iNKT cells. As shown in Figure 2, GSL-1 is an α*-*linked glycosylceramide containing a glucuronic saccharide and a sphingonine-based ceramide and GSL-1' is its galacturonic acid counterpart. Also found in this family of bacteria are higher order GSLs (GSL-3 and GSL-4), which differ primarily from GSL-1 by the presence of additional saccharides, including the presence of a glucosamine subunit in both. GSLs share remarkable similarities to α*-*GalCer, especially in the α*-*glycosyl linkage.

GSL-1 is not as strong of an iNKT cell antigen as α*-*GalCer. The difference in stimulatory activity of these two glycolipids likely results from protein interactions, which differ due to the stereochemistry at the C4"-position in the glycolipids (gluco *vs.* galacto). Crystal structures of the TCR-GSL1-CD1d and TCR-α*-*GalCer-CD1d complexes show different hydrogen-bonding networks, which result in a lateral shift of the galactosyl head group in α*-*GalCer relative to the glucuronosyl head group in GSL1. This lateral shift is thought to explain the difference in antigenicity between GSL-1 and α*-*GalCer [19,35,36].

As is customary in the practice of structural confirmation of isolated natural products, Long *et al.* [35] and Kinjo *et al.* [37] synthesized GSLs from *Sphingomonadaceae* to validate proposed structures and determine their stimulatory activity with iNKT cells. These studies confirmed the previously published evidence that GSL-1 is an iNKT cell antigen. However, it was observed that synthetic forms of the higher order GSLs, GSL-3 and GSL-4, were not strong antigens for iNKT cells. This observation was unexpected for two reasons: (1) additional sugars attached to α*-*GalCer are enzymatically removed in the lysosome revealing antigenic α*-*GalCer, and it was expected that a similar process would occur with the higher ordered GSLs revealing GSL-1, and (2) in an earlier report, [34] isolated GSL-4 was reported as a potent iNKT cell antigen. Using glycolipids where the sugars found in GSL-3 and GSL-4 were appended on α*-*GalCer, Long *et al.* [35] showed that lysosomal truncation of GSL-3 or GSL-4 to GSL-1 did not readily occur. Therefore, it is likely that the isolated GSL-4 was contaminated with GSL-1.

These conflicting results between synthetic and isolated glycolipids are not unique to GSL-4. For example, Fischer *et al.* [38] presented PIM4, a pentahexose phosphoinositol isolated from *Mycobacterium bovis bacillus*, as an exogenous NKT cell antigen [9]. However, two years later, Kinjo *et al.* [39] synthesized PIM4 and found that it did not stimulate NKT cells. These examples, among others, underscore the importance of comparisons of isolated and synthetic potential iNKT cell antigens.

### 2.2. Bacterial Diacylglycerols

The discovery that microbial glycolipids stimulate iNKT cells provided insight into the role of iNKT cells in innate immunity. However, it is generally accepted that bacteria from the *Sphingomonadaceae* family, while ubiquitous, are not common human pathogens. In 2006, Kinjo *et al.* [39] reported an iNKT cell antigen from a noted pathogenic bacterium, *Borrelia burgdorferi*, the causative agent of Lyme disease. Lyme disease affects an estimated 33,000 people each year in the U.S. making it the most common vector-borne disease in the U.S. [40]. After observing that iNKT cell knockout mice have a higher bacterial burden when infected with *B. burgdorferi* than wild-type mice, Kinjo *et al.* [39] demonstrated that iNKT cells were activated * in vivo* during an infection with this organism.

In the process of characterizing antigenic glycolipids in *B. burgdorferi*, a series of galactosyl diacylglycerols (DAGs) were isolated. These glycolipids contain varied lengths of acyl chains and different degrees of unsaturation. To further ensure that the DAGs were the active iNKT cell stimulatory components in the tested extracts, Kinjo *et al.* [39] synthesized a panel of DAGs of varying lipid compositions, and tested them on a variety of mouse and human iNKT cells. BbGL-II (Figure 2) stimulated the majority of the iNKT cells and, notably, was the first reported non-glycosphingolipid iNKT cell antigen. Four years later, Wang *et al.* [10] further characterized the structural requirements necessary for the binding of *B. burgdorferi* antigens to CD1d. Analysis of the crystal structure of many isoforms of DAGs bound to CD1d showed that the length and degree of saturation of the acyl chains, specifically which acyl chain is bound in the A' or F' pocket, impacts how the glycolipid is bound in CD1d. Furthermore, alternate binding motifs result in different orientations of the carbohydrate head group. The chain lengths and unsaturation found in BbGL-II provide a galactose orientation comparable to that found in the CD1d complex with α*-*GalCer.

In 2011, Kinjo *et al.* [41] presented another set of glycosylated DAG iNKT cell antigens isolated from the pathogen *Streptococcus pneumoniae*. These glycolipids are similar to the DAGs found in *B. burgdorferi* (Figure 2), and they further solidified the role of iNKT cells in recognizing bacterial pathogens. α*-*Linked glucosyl DAGs and a disaccharide Gal-Glu-DAG (Figure 2) were isolated, characterized, and tested for iNKT cell stimulatory activity. Interestingly, the glucosyl DAGs were found to be as potent as the purified synthetic versions of the galactosyl DAG glycolipids. This observation remains a distinguishing characteristic of DAG glycolipids from its GSL counterparts; with DAG-based glycolipids, galacto and gluco forms provide comparable iNKT cell stimulatory activity, while with glycosphingolipids, the galacto forms are more stimulatory than the corresponding gluco isomers. It is thought that the single double bond in the vaccenic lipid (sn-2) positions the DAG antigens so that their saccharide portion is presented in a tilted configuration with respect to α*-*GalCer.

### 2.3. Protozoan Phosphoinositols

In 2009, a phosphoinositol was isolated from the membranes of the pathogenic protozoan parasite *Entamoeba histolytica* and shown to stimulate iNKT cells [42]. It is estimated that *E. histolytica* causes 100,000 deaths per year worldwide making it second only to malaria in global parasitic morbidity [43]. Upon observing that iNKT cell knockout mice had significantly larger abscesses than the wild-type mice after being subjected to experimentally induced amebic liver infection [44], Lotter* et al.* [42] isolated lipopeptidophosphoglycan (LPPG) from *E. histolytica* trophozoites. Interestingly, The LPPG extract potently stimulated spleen and liver iNKT cells *in vitro*, manifested by the generation of IFN-γ, but not IL-4. Further isolation and characterization of the extract led to the discovery of the agonistic component of LPPG, a dilipid phosphoinositol antigen (EhPIb) (Figure 2). Their data suggested that the EhPIb directly stimulates iNKT cells. They also showed that iNKT cells are indirectly stimulated though IL-12 pathways.

### 2.4. Fungal Glycolipids

Over the past decade, substantial evidence has accumulated suggesting that iNKT cells are involved in triggering asthma. In addition, common molds have been associated with increased incidence of asthma. These observations prompted Umetsu and coworkers [45] to explore possible iNKT cell antigens in common molds, with an emphasis on *Aspergillus* spp. These fungi are logical targets since they have been reported to present significant respiratory exposure in humans [46,47]. Their labors proved fruitful as they reported the first fungal iNKT cell antigen, asperamide B (Figure 2). This group showed that *A. fumigatus* and *A. niger* extracts, isolated and purified asperamide B, and synthetic asperamide B rapidly induced airway hyperreactivity and airway inflammation, cardinal features of allergic asthma *in vitro* and *in vivo* [45]. Asperamide B was previously isolated and characterized from *A. niger* [48].

Unlike the previously described iNKT cell exogenous antigens, asperamide B (Figure 2) is a β*-*linked glycolipid. It also contains modified acyl and sphingosine chains, as compared to α*-*GalCer. The acyl chain is βδ unsaturated with an α hydroxyl group. The sphingosine chain is unsaturated with two additional methyl groups. These differences in structure may account for the recognition of this glycolipid as a β anomer rather than the more common α form seen with other iNKT cell antigens [49]. 

### 2.5. H. pylori Glycolipid

Supporting the recent report that *Helicobacter pylori*, a common Gram-negative bacterium found in the stomach, infections are inversely correlated with asthma [50], Chang *et al.* [51] found that administration of a cholesterol-based glycolipid (PI57 Figure 2) from *H. pylori* to suckling mice prevented allergen induced airway hyperreactivity later in their lifecycle. This compound was also shown to activate both mouse and human CD1d-restricted iNKT cells in a TH1-biased manner. 

## 3. Endogenous Antigens for iNKT Cells

During iNKT cell development in the thymus, the TCR of an iNKT cell population samples low affinity endogenous antigens as part of the positive selection process. If the TCR samples a high affinity ligand or if it does not sample an endogenous ligand, then the T cell population is clonally deleted [52]. Efforts have been made to identify and characterize these selecting endogenous antigens. Initially it was believed that there was a single endogenous antigen used during iNKT cell development, and that this antigen must be a glycosphingolipid, like the previously discovered iNKT antigens. Supported by work presented by Kronenberg and coworkers [53], this notion has started to shift towards the existence of several endogenous antigens and the possibility of non-glycosphingolipid antigens. Notably, Kronenberg and coworkers [53] tested multiple iNKT cell lines with diminished ability to synthesize glycosphingolipids (so that any glycosphingolipid endogenous antigens were eliminated). Surprisingly, these mutant cell lines still stimulated iNKT cells. In addition, the group utilized N-butyldeoxygalactononojirimycin, a selective inhibitor of ceramide glucosyltransferase, on autoreactive iNKT cell lines. These lines also retained the ability for the endogenous auto-reactive DC cells to stimulate iNKT cells. These data suggest the presence of endogenous iNKT cell antigens that are not glycosphingolipids. Similar to the exogenous antigens discussed a wide variety of endogenous iNKT cell antigens likely exist. Additional evidence for the presence of endogenous antigens for iNKT cells comes from the autoreactivity of iNKT cells. It has become increasingly clear that recognition of endogenous antigens by iNKT cells plays a key role in controlling immune responses. Multiple endogenous antigens for iNKT cells have been proposed; however, it is not yet fully understood how the production of endogenous antigens and subsequent activation of iNKT cells is regulated [3]. 

The first endogenous iNKT cell antigen reported was isoglobotrihexosylceramide (iGb3) [54]. iGb3 was implicated as an endogenous antigen from the observation that β*-*hexosaminadase knockout mice had a significant decrease in the number of iNKT cells. This enzyme is responsible for the truncation of GalNAc residues from a variety of lysosomal glycolipids yielding iGb3, isomeric Gb3, and other related glycosphingolipids. Synthesis of affected glycolipids and observation of iNKT cell stimulation by them led to the observation that among this group of glycolipids only iGb3 proved stimulatory [54]. The relevance of iGb3 as an iNKT cell antigen in humans is complicated by the reported lack of iGb3 synthase in humans (enzyme responsible for iGb3 synthesis) [55], the inability of human CD1d to present iGb3 to iNKT cells [56], and the lack of impact on iNKT cells in iGb3 synthase deficient mice [57,58]. Nevertheless, iGb3 remains an important candidate as an endogenous iNKT cell antigen due to its ability to stimulate iNKT cells [56,57].

**Figure 3:**
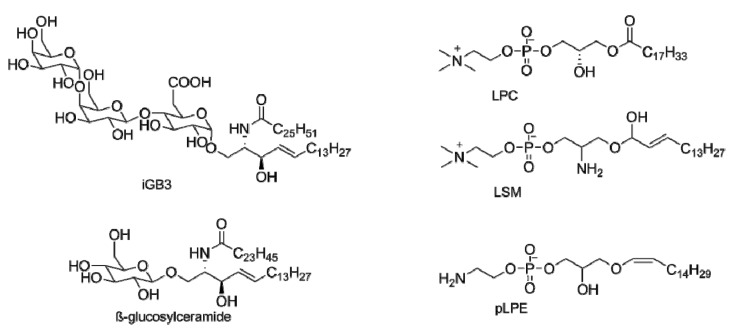
Proposed endogenous iNKT cell antigens.

In an effort to identify additional endogenous ligands, Cox *et al.* [59] performed a thorough isolation and characterization analysis of natural ligands eluted from CD1d. Among the eluted ligands, lysophosphotidylcholine (LPC) (and to a lesser extent lysosphingomelin (LSM), Figure 3) was found to stimulate some subsets of iNKT cells [60]. This was particularly interesting because of the reported increased frequency of LPC and LPC-specific T cells found in the plasma of melanoma patients [61]. Bound predominately in the F' pocket, LPC binds to CD1d with relatively low affinity [62]. Though LPC-reactive iNKT cells have been discovered, it remains to be established what fraction of iNKT cells are LPC-reactive.

In the continuing effort to identify endogenous antigens, Libero and coworkers [63] extracted and fractionated lipids from mouse thymocytes and tested their ability to stimulate freshly isolated iNKT cell thymocytes. Further purification via HPLC and characterization via tandem mass spectrometry identified two phosphoethanolamine endogenous iNKT cell antigens, namely eLPA and pLPE (Figure 3). These peroxisome-derived ether-bonded phosphoethanolamine compounds induced similar IL-4 production when compared to α*-*GalCer. GNPAT knockout mice, mice lacking a key enzyme necessary for the synthesis of these compounds, exhibit multiple abnormalities as well as decreased numbers in iNKT cells further supporting the importance of these lipids. GNPAT knockout mice had reduced numbers of iNKT cells further suggesting the presence of multiple selecting endogenous antigens for the maturation of iNKT cells.

Recently it has been reported that β*-*glucosylceramide (β*-*GluCer, Figure 3) is also an endogenous iNKT cell antigen. During a screening of naturally occurring glycosphingolipids, Brenner and coworkers [64] found that β*-*GluCer activated mouse and human iNKT cells. Earlier, Joyce and coworkers found that a deficiency in glucosylceramidase resulted in a decrease in the number of iNKT cells [65]. In confirming this finding, Brenner’s group found that enzymatically blocking the synthesis of β*-*GluCer resulted in a significant decrease in iNKT activity. In contrast to this report, Facciotti *et al.* [63]. found that non-oxidized self-glycosphingolipids did not stimulate iNKT cells. Therefore, additional research will be required to confirm the role of β*-*GluCer on iNKT cells.

## 4. α-GalCer Analogs

The presentation protein CD1d binds the lipid tails of glycosphingolipids and glycodiacylglycerols in the A' and F' pockets, interacts with the sphingosine portion of glycolipids, and presents the sugar head group to the TCRs on iNKT cells. Multiple research groups have investigated impacts of structural modifications at each of these three regions, and the influences of structural variation on iNKT cell responses have been determined.

### 4.1. Modification of the Lipid Chains

**Figure 4:**
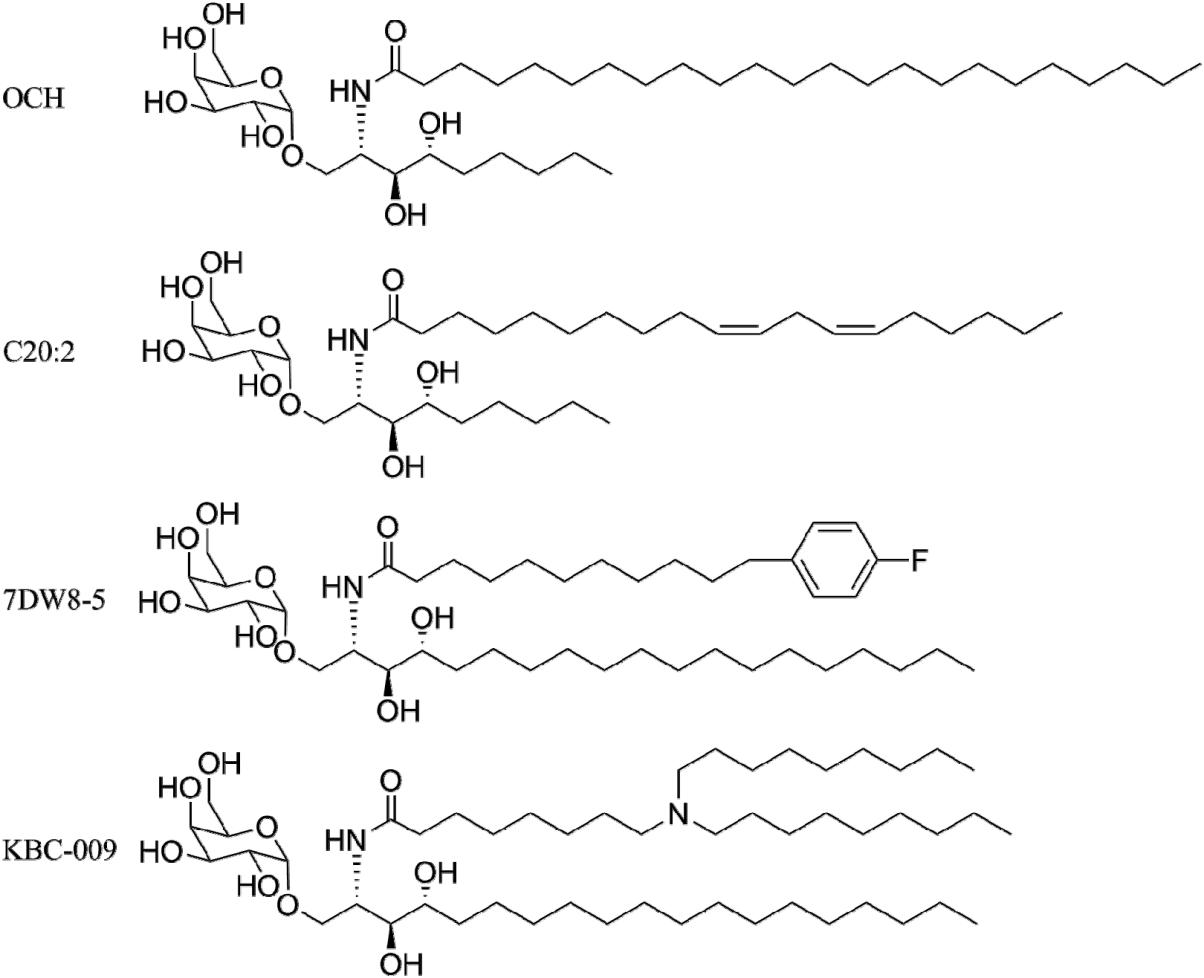
iNKT cell antigens with varied lipid tails.

After the discovery that α*-*GalCer was a CD1d ligand, [27] it was realized that α*-*GalCer might not be as therapeutically useful as possible due to the secretion of counteracting immunostimulatory and immunomodulatory cytokines and the observed iNKT cell anergy upon stimulation with α*-*GalCer [12]. This provided an opportunity for researchers to develop analogs that could bias iNKT cell stimulation to either a TH1 or TH2 response. Yamamura and coworkers [66] synthesized analogs with modified phytosphingosine moieties in an attempt to bias the cytokine release profile of iNKT cells. Although the analogs were less potent than α*-*GalCer, one analog, having a shortened phytosphingosine lipid chain (OCH, Figure 4), displayed a biased TH2 iNKT cell response. In addition, it was reported that administration of OCH effectively suppressed experimental autoimmune encephalomyelitis (a mouse model for multiple sclerosis). Shortly after this report, another TH2 biased analog, glycolipid C20:2 (Figure 4), was identified [67]. This analog stimulated iNKT cells more potently than OCH, but still less potently than α*-*GalCer. Additionally, Forestier *et al.* [68] reported that OCH was unable to stain human iNKT cells, whereas the C20:2 analog showed significant staining. These results, along with the observation that NOD mice treated with the C20:2 analog had a prolonged survival rate and lower incidence of diabetes, has made C20:2 a potential therapeutic target for type 1 diabetes. In an effort to develop an even stronger TH2 antigen, Velmourougane *et al.* [69] designed an antigen that combined the acyl chain of glycolipid 20:2 and the phytosphingosine lipid chain length of OCH. This analog stimulated iNKT cells in a TH2 biased manner, but was less active than both parent analogs; therefore, it offered no advantage over either analog.

These reports support the fact that small modifications of the lipid tails of glycolipids can substantially impact iNKT cell stimulation. To establish the effects of lipid-chain lengths on iNKT cell activation, Goff *et al.* [70] synthesized a library of α*-*GalCer analogs that varied by lipid lengths of the acyl and sphingosine tails of α*-*GalCer. Measuring the ratio of IFN- γ and IL-4 to quantify TH1 *vs.* TH2 bias, a clear correlation between lipid chain lengths and iNKT cell stimulation was formed. The shorter the sphingosine or acyl chain length, the more IL-4 was produced consequently biasing a TH2 response. Oki *et al.* [71] hypothesized that IFN- γ production requires longer TCR stimulation than IL-4 production. The shorter lipid chain length analogs do not form as stable complexes with CD1d than the longer chain analogs, due to the lack of additional hydrophobic interactions in the A' or F' pockets. These short-chain analogs stimulate the TCR for a short amount of time resulting in primarily IL-4 production.

Wong and coworkers [72] developed analogs designed to enhance the interactions between α*-*GalCer and the lipid-binding pockets of CD1d, which ultimately have led to potent TH1 biasing iNKT cell antigens. In 2005, they reported the synthesis of multiple α*-*GalCer analogs that differed in the composition of their acyl lipid chains. α*-*GalCer analogs containing aromatic moieties attached to their acyl chains expanded iNKT cells more potently than α*-*GalCer and displayed a marked TH1 biased cytokine profile. Additionally, these analogs exhibited more potent anti-cancer activity than α*-*GalCer in lung and breast cancer models [73]. These analogs demonstrated up to an 80-fold higher binding affinity to CD1d than α*-*GalCer. It was proposed that the higher binding affinity is due to a tighter fit in the binding groove, by the bulky phenyl substituents, making the association more entropically favored. Crystal structures were solved and helped elucidate the molecular basis for a skewed TH1 response. Additional hydrogen bonds from the phenyl group were also observed likely adding to the increase in binding affinity [74]. Further development of aromatic-containing TH1 analogs has led to promising vaccine adjuvants results, with 7DW8-5 (Figure 4) exhibiting the most potent adjuvant activities [75].

The successful design of TH1 and TH2 biased iNKT cell antigens via modification of the lipid tails of α*-*GalCer has guided the design of recently reported iNKT cell antigens. It has been shown that ether containing lipids [76,77], fluorinated lipids [78], and N-acyl variants (e.g., halogenated phenyl groups, adamantonyl groups) [79] are well tolerated. Notably, Lee *et al.* [80] synthesized branched acyl chain α*-*GalCer analogs (Figure 4) to increase the solubility of α*-*GalCer, a possible clinical limitation of α*-*GalCer. Additionally they showed that their analogs had potent adjuvant properties demonstrated by protection from a nasal influenza challenge after co-administration with the vaccine.

### 4.2. Modifications of the Sphingosine Base

To better understand interactions of CD1d with the sphingosine portion of glycosphingolipids, diastereomers that differ between the 3'-and 4'-hydroxyls were synthesized to explore the impact of these hydroxyls in the context of anti-tumor activity [26]. These diastereomers and other analogs from the first SAR study of agelasphins demonstrated that the 3'-hydroxyl group was necessary for anti-tumor properties, with the 4'-hydroxyl having little to no impact on the anti-tumor potency [25]. During the following years, others have modified the phytosphingosine base to elucidate and confirm the role of the 3'- and 4'-hydroxyl on iNKT cell stimulation, as well as modify the amide of the phytosphingosine base as a means to find therapeutic iNKT cell antigens.

**Figure 5:**
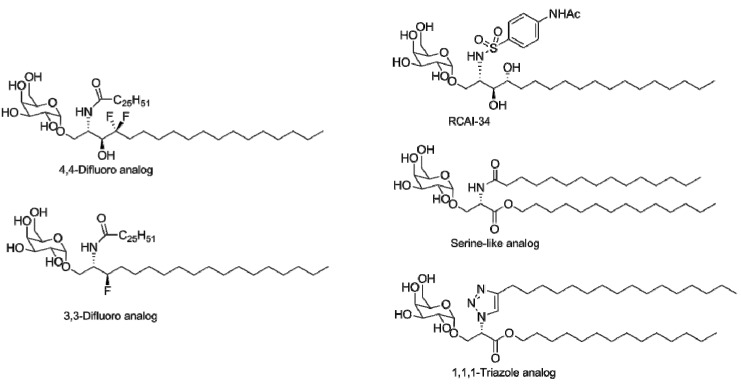
iNKT cell antigens with varied sphingosine structures.

After the crystal structure of the TCR- α*-*GalCer-CD1d complex was published, a renewed interest developed over the roles of the phytosphingosine scaffold of α*-*GalCer, in particular the 3'- and 4'-hydroxyls. It was shown that three hydrogen bonds help dock and orient α*-*GalCer for presentation to the TCR. One of these crucial hydrogen bonds involves the 3'-hydroxyl [79]. The crystal structure does not show any meaningful interaction with the 4'-hydroxyl, and this observation has also been supported by the relatively similar iNKT cell potency of the sphinganine and sphingosine analogs [81,82,83]. To further test the importance of the 3'- and 4'-hydroxyls (Figure 5), Leung* et al.* [84] installed two flourines to replace the 4'-hydroxyl. This analog stimulated iNKT cells, further validating the claim that the 4'-hydroxyl is of little importance. Trappeniers* et al.* [82] and Park *et al.* [85] synthesized and evaluated α*-*GalCer epimers that differed by the stereochemistry of the hydroxyls in question as well as the amide. It was found that the stereochemistry at the 2' and 3' positions is crucial, and that the stereochemistry at the 4' position is not important for the stimulation of iNKT cells.

Recently, additional conflicting reports have been published over the role of the 4'-hydroxyl. In 2012, the sphingosine, sphingonine, and phytosphingosine versions of α*-*GalCer were reported to stimulate mouse and human iNKT cells differently [86]. That is that the three compounds all activated mouse iNKT cells similarly, but in contrast, the human iNKT cell stimulation was significantly lower for the sphingosine and sphingonine compounds (observed in two separate human iNKT cell assays), suggesting an important role of the 4'-hydroxyl in human iNKT cell stimulation. For the case of the 4*l*-hydroxyl, Hunault *et al.* [87] have continued to explore and confirm the role of the 4'-hydroxyl. This group synthesized fluorinated 3',4'-dideoxy α*-*GalCer analogs. In support of their previous data, it was shown that the 4'-deoxy α*-*GalCer analog is an iNKT cell agonist with murine and human iNKT cells.

While the roles of the 3'- and 4'-hydroxyls have been thoroughly explored, studies have also been reported on the modification of the phytosphingosine amide. In 2007, Lee* et al.* [88] replaced the amide with a 1,2,3-triazole moiety (Figure 5). Their rationale for this modification is the stability of triazoles to enzymatic hydrolysis, which might impart a more profound stimulatory response. They reported that the triazole moiety was tolerated and resulted in a TH1 cytokine release profile. Esters [89], ethers [89,90], sulfonamides [91], and primary amines [90] have also been used as amide replacements. The ether and amine analogs did not induce iNKT cell stimulation, suggesting that the hydrogen bond to the amide nitrogen is essential for iNKT cell stimulation [89,90]. The ester analogs induced a TH2 cytokine response, albeit less potently than α*-*GalCer [89]. The sulfonamide analogs also biased a TH2 response *in vitro*, but failed to elicit iNKT cell proliferation *in vivo* [91] Serine-based ceramide analogs have also been explored [92]; these stimulated iNKT cells similarly to α*-*GalCer, but were significantly less potent. Recently, Sun* et al.* [90] designed analogs that effectively tested the importance of the positional requirement of the galactose by introducing a short linker in between the ceramide and the glycosidic bond, as well as an analog that attached the saccharide at the 2'-position. These analogs displayed no detectable iNKT cell activation. All in all, the recent data suggests that the hydrogen bond to the amide nitrogen or a suitable replacement is mandatory for iNKT cell stimulation and the positional requirement of the galactose for TCR activation is crucial.

### 4.3. Modifications of the Glycosidic Linkage

**Figure 6:**
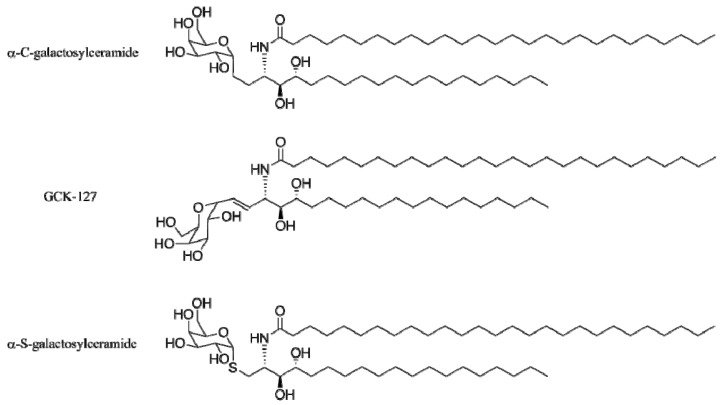
C-glycoside antigens for iNKT cells.

In an attempt to develop more potent iNKT cell antigens, groups have also modified the glycosidic linkage of α*-*GalCer. The first and most promising candidate was a carbon-containing glycosidic bond analog, α*-*C-GalCer (Figure 6). Administration of this analog demonstrated enhanced protection of malaria in mice (in comparison to α*-*GalCer), as well as enhanced prevention of melanoma metastasis in mice. Because both of these disease states are related to the release of IFN-γ, these results suggested that α*-*C-GalCer was a better inducer of IFN-γ than α*-*GalCer. As expected, the cytokine release profile after α*-*C-GalCer administration resulted in a highly skewed TH1 bias of cytokines [93]. Over the years, this compound has proven to be a better or comparable iNKT cell agonist than α*-*GalCer in multiple disease state models (*i.e.*, arthritis, asthma, melanoma) [94] and as a potential vaccine adjuvant [95]. 

To understand the therapeutic activity of α*-*C-GalCer and to possibly enhance its activity, several groups have designed analogs of α*-*C-GalCer. Analysis of the α*-*GalCer-CD1d and TCR-α*-*GalCer- CD1d crystal structures provided two relevant features. First, the glycosidic oxygen participates in hydrogen-bonding which would be absent in the α*-*C-GalCer-CD1d complex. This would result in weaker binding to the CD1d-TCR complex. Second, α*-*GalCer is presented at a 170 degree dihedral angle, and this might be an optimal angle for presentation to the TCR interface [81,96]. These features prompted Franck and coworkers [97] to test the optimal conformation of the glycosidic bond by synthesizing α*-*C-GalCer analogs with a double bond linking the anomeric carbon of galactose to ceramide, effectively locking the sugar in place (GCK-127, Figure 6). The *E* unsaturated analog was shown to induce greater amounts of IL-12 and lesser amounts of IFN-γ than α*-*C-GalCer. These results guided Li *et al.* [98] to optimize the *E*-alkene analogs. Many of the tested analogs differed in their cytokine profiles in a species-specific manner, with the exception of GCK-127; it was able to stimulate mouse and human iNKT cells more efficiently than α*-*C-GalCer. These effects on iNKT cell stimulation, coupled with the observation that α*-*C-GalCer has a much lower binding affinity to the NKT cell TCR than α*-*GalCer-CD1d tetramers [99], suggested that α*-*C-GalCer was being presented to iNKT cells significantly differently than α*-*GalCer. To further understand these differences, Patel *et al.* [100] solved the TCR-α*-*C-GalCer-CD1d crystal structure. Not surprisingly, a key difference between the α*-*GalCer and α*-*C-GalCer docking modes was a lost hydrogen bonding interaction; this seems to shift the galactose so that it is presented at a higher position to the TCR, which in turn weakens the α*-*C-GalCer-TCR contacts at the sugar position. Although this explains the lower affinity of α*-*C-GalCer, it does not explain the aforementioned *in vivo* and *in vitro* activity of α*-*C-GalCer. This underscores the fact that TCR affinity does not always correlate with iNKT cell activity.

The second set of α*-*GalCer analogs that differ by their glycosidic bond are the thio-glycoside derivatives. In 2007, Chang *et al.* [73] synthesized and evaluated 16 analogs of α*-*GalCer. The analogs mostly varied between the composition of their acyl and phytosphingosine chains. Among these analogs was an analog that replaced the oxygen glycosidic bond with a sulfur bond (α*-*S-GalCer, Figure 6). This α*-*S-GalCer induced a slight expansion of human iNKT cells *in vitro* and exhibited a nominal TH2 cytokine release profile, but was not selected in the study as a viable iNKT cell agonist because of its poor anticancer efficacy. Blauvelt *et al.* [101] and Dere *et al.* [102] envisioned the sulfur-carbon bond being beneficial for two reasons: (1) thiol glycosidic bonds are more resistant to enzymatic cleavage, and (2) the longer carbon-sulfur bond might position the galactose into a more favorable position for iNKT cell stimulation. These two groups simultaneously synthesized and analyzed α*-*GalCer for iNKT cell stimulation. Blauvelt *et al.* [101] reported that α*-*S-GalCer could not induce iNKT cell proliferation or cytokine secretion of mouse iNKT cells *in vitro* and *in vivo*. Three years later, Hogan *et al.* [103] presented their immunological results. They demonstrated that α*-*S-GalCer stimulates human iNKT cells *in vitro*, but, in agreement with the previous studies, α*-*S-GalCer did not stimulate mouse iNKT cells *in vivo*.

### 4.4. Modifications of the Saccharide

After the discovery of the potent anti-tumor activities of α*-*GalCer, Kirin Breweries continued to explore the structure-activity relationships of the saccharide head group. They found, by testing a series of disaccharides, that the modifications at the 2"-, 3"-, and 4"-positions diminished the anti-tumor properties [27]. Since these observations, other groups have confirmed these studies through the design and synthesis of 2"-α*-*GalCer analogs (e.g., 2"-fluoro [104], 2"-deoxy [104], and 2"-methoxy analogs [105]). With the notable exception of a haptenated 2"-hydroxyl-α*-*GalCer analog designed to study the mechanism of anti-lipid antibody production [106], most major modifications at the 2"-position have resulted in a loss of stimulatory activity. The importance of the 3"- and 4"-hydroxyls has also been explored by the immunological evaluation of 3"-sulfated glycolipids [107], 3"- and 4"-deoxy and -fluoro analogs [108], and a panel of 3"- and 4"-analogs (synthesized by Wong and coworkers) [109,110]. Unlike the 2"-hydroxyl analogs, small modifications at the 3"- and 4"-positions are moderately tolerated, albeit with a decrease in stimulatory activity.

These observations were later explained from the crystal structure of the iNKT cell TCR-α*-*GalCer-CD1d crystal structure [81]. The 2"-, 3"-, and 4"-hydroxyls make hydrogen bonds with amino acids in the TCR of iNKT cells, whereas the 6"-hydroxyl is exposed to the solvent. A non-glycosidic α*-*GalCer analog probed the ability to minimize the amount of contacts necessary for stimulation. ThrCer, a ceramide with an α-threitol chain, was able to stimulate iNKT cells without the associated iNKT cell anergy. This analog makes the necessary hydrogen-bonds with the TCR of iNKT cells, further providing evidence for the importance of the hydrogen bonds in iNKT cell stimulation [111]. 

#### 4.4.1. 6"-α*-*GalCer Analogs

Before the availability of the crystal structure of α*-*GalCer bound to CD1d, few things were known about the structural requirements necessary for iNKT cell activation. Kronenberg and coworkers [112] showed that a Gal(α1-6)GalCer glycolipid did not require lysosomal processing for iNKT cell activation, suggesting that modification at the 6" position was well tolerated. These results, among others, were later explained by analysis of the crystal structure of the bound glycolipid. The crystal structures provided clear evidence that the 6"-hydroxyl is positioned away from the TCR-CD1d interface, thus explaining why 6"-modified analogs were tolerated. Because of this tolerance, 6"-α*-*GalCer analogs have been an attractive target in the design of variants with diverse applications and properties.

**Figure 7:**
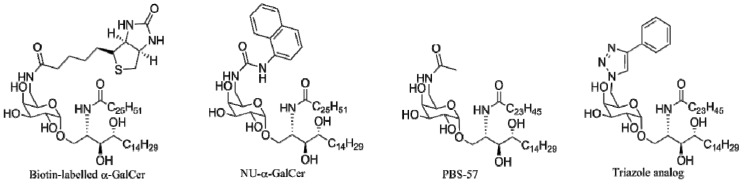
6"-α-GalCer analogs for iNKT cells

One such application was demonstrated by Zhou* et al.* [113], who converted the 6"-hydroxyl to an amine group, allowing for the coupling of fluorophores (e.g., dansyl and prodan) and biotin (Figure 7). These analogs were designed to be used as tools in studying the trafficking, processing, and presentation of glycolipids. These analogs further provided evidence that the 6"-hydroxyl position was not necessary for iNKT cell stimulation, since they stimulated iNKT cells comparably to α*-*GalCer. Xia* et al.* [114] and Cheng* et al.* [115] reported flow cytometry data for the biotin and dansyl labeled analogs, respectively. These reports demonstrated that these 6"-α*-*GalCer analogs can be used as probes for the analysis of glycolipid uptake. One possible issue with these analogs is that modifying the glycolipid with relatively large fluorophores or biotin might interfere with or completely change the trafficking, processing, and presentation. To address these possible issues, Liu* et al.* [116] synthesized Gal(α1-2)αGalCer and compared it with Gal(α1-2)PBS-57, a 6"-deoxy-6"-acetamide α*-*GalCer analog, in a lysosomal processing model. Their studies concluded that the acetamide position did not affect lysosomal processing. The effect of the 6"-modification towards trafficking and presentation has not been fully elucidated.

6"-Analogs have also been designed to overcome the therapeutic and practical limitations of α*-*GalCer. For example, α*-*GalCer’s therapeutic effectiveness is limited by the observed iNKT cell anergy and counteracting released cytokines, but it has practical limitations because of its poor solubility in organic and aqueous solvents. To address this issue, Liu* et al.* [117] incorporated an acetamide functional group at the 6"-position to increase the solubility of α*-*GalCer (PBS-57 Figure 7). The increased solubility of PBS-57 in DMSO not only facilitated handling, but also imparted an increase in iNKT cell stimulation* in vivo* and* in vitro* when compared to α*-*GalCer.

The increased solubility and stimulatory activity of PBS-57 prompted Trappeniers *et al.* [118] to design a small set of related 6"-analogs. Using a precursor of PBS-57, they attached amide- and urea-linked aromatic functionalities with varying steric and electronic properties. Many of the analogs provided a TH1-biased cytokine-release profile, with the promising analog Nu-α*-*GalCer (Figure 7), a urea linked naphthyl analog, producing more IFN-γ than α*-*GalCer accompanied with a diminished production of IL-4. This marked the first sugar-modified analog that could bias the iNKT cell response. Aspeslagh *et al.* [119] further studied these analogs to explain the observed phenomena. They presented the mouse CD1d-Nu-α*-*GalCer-TCR crystal structure. This revealed that the naphthyl functional group of NU-α*-*GalCer sat in a small induced-fit binding pocket above the A' pocket instead of protruding into the solvent. In addition to a hydrogen bond interaction from the carbonyl oxygen from the urea substituent, the induced-fit binding pocket imparts a higher affinity of NU-α*-*GalCer to CD1d resulting in a longer interaction with iNKT cells and a TH1 biased response. Aspeslagh *et al.* [119] also presented data that NU-α*-*GalCer provided superior prevention of tumor metastasis in B16 mice when compared to α*-*GalCer.

Similar to the 6"-amide and urea analogs, Jervis* et al.* [120] designed a series of 6"-triazole α*-*GalCer analogs (Figure 7). Using click chemistry they were able to efficiently synthesize triazole-linked aryl and acyl substituents at the 6"-position. Similar to the amide- and urea-linked analogs, the triazole-linked compounds demonstrated biasing towards either a TH1 and, in one case, a TH2 response (although less pronounced). These results show that subtle differences at the 6"-position can impart large differences in iNKT cell stimulation. Although a promising candidate was not obtained, this report indicated that the triazole moiety was well tolerated. Jervis* et al.* [121] utilized this method to form homodimeric α*-*GalCer analogs in an attempt to mirror the reported higher affinity of multimeric ligands in other biological systems. The analogs stimulated iNKT cells but did not significantly improve upon the immunological activity of α*-*GalCer.

#### 4.4.2. Carbocyclic Analogs of α*-*GalCer

**Figure 8:**
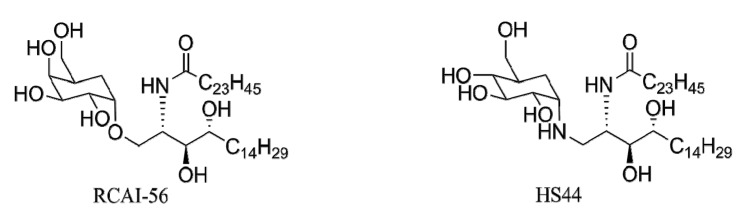
Carbocyclic analogs of α-GalCer.

Another saccharide modification that has received significant attention has been the design of carbo- cyclic analogs of α*-*GalCer. In order to explore the importance of the oxygen atom in the pyranose ring, Tashiro *et al.* [122] synthesized the carbocyclic versions of α*-*GalCer (RCAI-56, Figure 8). Preliminary bioassays of RCAI-56 showed a significant increase in IFN-γ when compared to α*-*GalCer. The follow up immunological evaluation of RCAI-56 confirmed that RCAI-56 is a strong TH1 iNKT cell agonist that increases IFN-γ production through increased IL-12 production [123]. Similar to α*-*C-GalCer, RCAI-56 benefits from an ether linkage instead of the acetal glycosidic bond resulting in less susceptibility towards enzymatic degradation. Unlike α*-*C-GalCer, RCAI-56 maintains the hydrogen bonding capabilities of the glycosidic bond towards binding to CD1d [123]. The strong TH1 response of RCAI-56 resulted in inhibiting the induction of collagen induced arthritis in mice, making this analog a potential therapeutic agent [124].

Similar to RCAI-56, Harrak *et al.* [125] synthesized a carbocyclic analog of α*-*GalCer, aminocyclitol HS44 (Figure 8). This analog was able to induce iNKT proliferation *in vitro* and displayed a similar cytokine release profile than OCH, albeit less potently. Unexpectedly, HS44 induced similar levels of IFN-γ to α*-*GalCer with nearly baseline levels of other cytokine released *in vivo.* This *in vivo* TH1 response was supported by the ability of HS44 to efficiently induce an antitumor response in B16 mice. Structural analysis of the mCD1d-HS44-iNKT TCR complex revealed diminished interactions with the TCR, accounting for the decrease in potency and binding affinity of HS44. This report is another example that binding affinity does not have a direct correlation to proliferation [126]. 

## 5. Conclusions

Identification of natural antigens has focused on endogenous and exogenous antigens, and it is likely that many of these have yet to be discovered. iNKT cell selection and iNKT cell autoreactivity argue for endogenous antigens, and these may play a key roles in regulating both innate and adaptive immune responses. A complete understanding of the structures and functions of endogenous antigens will likely allow manipulation of their production as a means of indirectly controlling iNKT cell activation. Exogenous antigens have been found in bacterial, fungal, and parasitic pathogens. A relatively small subset of organisms has been surveyed for iNKT cell antigens, and the search for exogenous antigens promises to continue to be a fruitful avenue of research.

The structures of designed glycolipid antigens for NKT cells can be conceptually divided into four portions: lipid chains, sphingosine/ceramide head group, the glycosidic bond, and the nature of the sugar. Structure-activity studies with glycolipids and iNKT cells have focused primarily on controlling the cytokine release profiles of iNKT cells in response to glycolipid stimulation. Multiple classes of glycolipid antigens effectively trigger TH1 responses from iNKT cells, and these responses have proven effective in adjuvanting vaccines and treating some forms of cancer. It is likely that these inflammatory responses would also beneficially impact responses to infection. Fewer antigens have been discovered that trigger TH2, or immunomodulatory responses. Activation of iNKT cells by this type of antigen may be generally useful in decreasing inflammation and ameliorating autoimmune diseases.

For years, the paradigm of antigen recognition by T cells involved MHC and peptide antigens. The discovery that a subset of T cells recognized glycolipids opened a new area for discovery and possible manipulation of immune responses. Observations of the roles that iNKT cells play in controlling bacterial, fungal, parasitic and viral infections and in influencing autoimmune diseases has heightened interest in natural and synthetic antigens for these cells. Research in this area has involved productive collaborations between synthetic organic chemists and immunologist and has resulted in discoveries of a series of natural and synthetic antigens for NKT cells. Continued collaborations between chemists and immunologists in this field will likely provide glycolipid antigens for iNKT cells, and potential applications range from use in improving the effectiveness of vaccines to treatment of infection to elimination of tumors to suppressing autoimmune diseases. 

## References

[1] Bendelac A., Savage P.B., Teyton L. (2007). The biology of NKT cells. Annu. Rev. Immunol..

[2] Savage P.B., Teyton L., Bendelac A. (2006). Glycolipids for natural killer T cells. Chem. Soc. Rev..

[3] Gapin L., Godfrey D.I., Rossjohn J. (2013). Natural killer T cell obsession with self-antigens. Curr. Opin. Immunol..

[4] Pellicci D.G., Patel O., Kjer-Nielsen L., Pang S.S., Sullivan L.C., Kyparissoudis K., Brooks A.G., Reid H.H., Gras S., Lucet I.S.;et al. (2009). Differential recognition of CD1d-alpha-galactosyl ceramide by the v beta 8.2 and v beta 7 semi-invariant NKT T cell receptors. Immunity.

[5] Zajonc D.M., Kronenberg M. (2007). Cd1 mediated T cell recognition of glycolipids. Curr. Opin. Struct. Biol..

[6] Cerundolo V., Barral P., Batista F.D. (2010). Synthetic iNKT cell-agonists as vaccine adjuvants--finding the balance. Curr. Opin. Immunol..

[7] Hong S., Scherer D.C., Singh N., Mendiratta S.K., Serizawa I., Koezuka Y., van Kaer L. (1999). Lipid antigen presentation in the immune system: Lessons learned from CD1d knockout mice. Immunol. Rev..

[8] Berkers C.R., Ovaa H. (2005). Immunotherapeutic potential for ceramide-based activators of iNKT cells. Trends Pharmacol. Sci..

[9] Wu D., Fujio M., Wong C.H. (2008). Glycolipids as immunostimulating agents. Bioorg. Med. Chem..

[10] Wang J., Li Y., Kinjo Y., Mac T.T., Gibson D., Painter G.F., Kronenberg M., Zajonc D.M. (2010). Lipid binding orientation within CD1d affects recognition of borrelia burgorferi antigens by NKT cells. Proc. Natl. Acad. Sci. USA.

[11] Cernadas M., Cavallari M., Watts G., Mori L., de Libero G., Brenner M.B. (2010). Early recycling compartment trafficking of CD1a is essential for its intersection and presentation of lipid antigens. J. Immunol..

[12] Cerundolo V., Silk J.D., Masri S.H., Salio M. (2009). Harnessing invariant NKT cells in vaccination strategies. Nat. Rev. Immunol..

[13] Godfrey D.I., Pellicci D.G., Patel O., Kjer-Nielsen L., McCluskey J., Rossjohn J. (2010). Antigen recognition by CD1d-restricted NKT T cell receptors. Semin Immunol..

[14] Zajonc D.M., Maricic I., Wu D., Halder R., Roy K., Wong C.H., Kumar V., Wilson I.A. (2005). Structural basis for CD1d presentation of a sulfatide derived from myelin and its implications for autoimmunity. J. Exp. Med..

[15] Kronenberg M. (2005). Toward an understanding of NKT cell biology: Progress and paradoxes. Annu. Rev. Immunol..

[16] Lawson V. (2012). Turned on by danger: Activation of CD1d-restricted invariant natural killer T cells. Immunology.

[17] Matsuda J.L., Mallevaey T., Scott-Browne J., Gapin L. (2008). Cd1d-restricted iNKT cells, the ‘swiss-army knife’ of the immune system. Curr. Opin. Immunol..

[18] Van Kaer L. (2005). Alpha-galactosylceramide therapy for autoimmune diseases: Prospects and obstacles. Nat. Rev. Immunol..

[19] Kinjo Y., Ueno K. (2011). iNKT cells in microbial immunity: Recognition of microbial glycolipids. Microbiol. Immunol..

[20] Schneiders F.L., Scheper R.J., von Blomberg B.M.E., Woltman A.M., Janssen H.L.A., van den Eertwegh A.J.M., Verheul H.M.W., de Gruijl T.D., van der Vliet H.J. (2011). Clinical experience with alpha-galactosylceramide (krn7000) in patients with advanced cancer and chronic hepatitis b/c infection. Clin. Immunol..

[21] Tsuji M. (2006). Glycolipids and phospholipids as natural CD1d-binding NKT cell ligands. Cell. Mol. Life Sci..

[22] Natori T., Koezuka Y., Higa T. (1993). Agelasphins, novel alpha-galactosylceramides from the marine sponge agelas-mauritianus. Tetrahedron Lett..

[23] Natori T., Morita M., Akimoto K., Koezuka Y. (1994). Agelasphins, novel antitumor and immunostimulatory cerebrosides from the marine sponge agelas-mauritianus. Tetrahedron.

[24] Kobayashi E., Motoki K., Yamaguchi Y., Uchida T., Fukushima H., Koezuka Y. (1996). Enhancing effects of alpha-,beta-monoglycosylceramides on natural killer cell activity. Bioorg. Med. Chem..

[25] Morita M., Motoki K., Akimoto K., Natori T., Sakai T., Sawa E., Yamaji K., Koezuka Y., Kobayashi E., Fukushima H. (1995). Structure-activity relationship of alpha-galactosylceramides against b16-bearing mice. J. Med. Chem..

[26] Morita M., Natori T., Akimoto K., Osawa T., Fukushima H., Koezuka Y. (1995). Syntheses of alpha-monoglycosylceramides, beta-monoglycosylceramides and 4 diastereomers of an alpha-galactosylceramide. Bioorg. Med. Chem. Lett..

[27] Kawano T., Cui J.Q., Koezuka Y., Toura I., Kaneko Y., Motoki K., Ueno H., Nakagawa R., Sato H., Kondo E. (1997). Cd1d-restricted and TCR-mediated activation of v(alpha)14 NKT cells by glycosylceramides. Science.

[28] Yu K.O.A., Porcelli S.A. (2005). The diverse functions of CD1d-restricted NKT cells and their potential for immunotherapy. Immunol. Lett..

[29] Matsuda J.L., Gapin L., Baron J.L., Sidobre S., Stetson D.B., Mohrs M., Locksley R.M., Kronenberg M. (2003). Mouse v alpha 14i natural killer T cells are resistant to cytokine polarization *in vivo*. Proc. Natl. Acad. Sci. USA.

[30] Uldrich A.P., Crowe N.Y., Kyparissoudis K., Pellicci D.G., Zhan Y.F., Lew A.M., Bouillet P., Strasser A., Smyth M.J., Godfrey D.I. (2005). NkT cell stimulation with glycolipid antigen *in vivo*: Costimulation-dependent expansion, bim-dependent contraction, and hyporesponsiveness to further antigenic challenge. J. Immunol..

[31] Parekh V.V., Wilson M.T., Olivares-Villagomez D., Singh A.K., Wu L., Wang C.R., Joyce S., van Kaer L. (2005). Glycolipid antigen induces long-term natural killer T cell anergy in mice. J. Clin. Invest..

[32] Mattner J., DeBord K.L., Ismail N., Goff R.D., Cantu C., Zhou D.P., Saint-Mezard P., Wang V., Gao Y., Yin N. (2005). Exogenous and endogenous glycolipid antigens activate NKT cells during microbial infections. Nature.

[33] Kinjo Y., Wu D., Kim G.S., Xing G.W., Poles M.A., Ho D.D., Tsuji M., Kawahara K., Wong C.H., Kronenberg M. (2005). Recognition of bacterial glycosphingolipids by natural killer T cells. Nature.

[34] Sriram V., Du W.J., Gervay-Hague J., Brutkiewicz R.R. (2005). Cell wall glycosphingolipids sphingomonas paucimobilis are CD1d-specific ligands for NKT cells. Eur. J. Immunol..

[35] Long X.T., Deng S., Mattner J.C., Zang Z., Zhou D., McNary N., Goff R.D., Teyton L., Bendelac A., Savage P.B. (2007). Synthesis and evaluation of stimulatory properties of sphingomonadaceae glycolipids. Nat. Chem. Biol..

[36] Wu D., Zajonc D.M., Fujio M., Sullivan B.A., Kinjo Y., Kronenberg M., Wilson I.A., Wong C.H. (2006). Design of natural killer T cell activators: Structure and function of a microbial glycosphingolipid bound to mouse CD1d. Proc. Natl. Acad. Sci. USA.

[37] Kinjo Y., Pei B., Bufali S., Raju R., Richardson S.K., Imamura M., Fujio M., Wu D., Khurana A., Kawahara K. (2008). Natural sphingomonas glycolipids vary greatly in their ability to activate natural killer T cells. Chem. Biol..

[38] Fischer K., Scotet E., Niemeyer M., Koebernick H., Zerrahn J., Maillet S., Hurwitz R., Kursar M., Bonneville M., Kaufmann S.H.E. (2004). Mycobacterial phosphatidylinositol mannoside is a natural antigen for CD1d-restricted T cells. Proc. Natl. Acad. Sci. USA.

[39] Kinjo Y., Tupin E., Wu D., Fujio M., Garcia-Navarro R., Benhnia M.R., Zajonc D.M., Ben-Menachem G., Ainge G.D., Painter G.F. (2006). Natural killer T cells recognize diacylglycerol antigens from pathogenic bacteria. Nat. Immunol..

[40] Centers for disease control and prevention (2013). Summary of Notifiable Diseases – United States 2011. Morb. Mortal. Wkly. Rep..

[41] Kinjo Y., Illarionov P., Vela J.L., Pei B., Girardi E., Li X.M., Li Y.L., Imamura M., Kaneko Y., Okawara A. (2011). Invariant natural killer T cells recognize glycolipids from pathogenic gram-positive bacteria. Nat. Immunol..

[42] Lotter H., Gonzalez-Roldan N., Lindner B., Winau F., Isibasi A., Moreno-Lafont M., Ulmer A.J., Holst O., Tannich E., Jacobs T. (2009). Natural killer T cells activated by a lipopeptidophosphoglycan from entamoeba histolytica are critically important to control amebic liver abscess. PLoS Pathog..

[43] (1997). Amoebiasis. Weekly Epidemiology Record.

[44] Lotter H., Jacobs T., Gaworski I., Tannich E. (2006). Sexual dimorphism in the control of amebic liver abscess in a mouse model of disease. Infect. Immun..

[45] Albacker L.A., Chaudhary V., Chang Y.J., Kim H.Y., Chuang Y.T., Pichavant M., Dekruyff R.H., Savage P.B., Umetsu D.T. (2013). Invariant natural killer T cells recognize a fungal glycosphingolipid that can induce airway hyperreactivity. Nat. Med..

[46] Shelton B.G., Kirkland K.H., Flanders W.D., Morris G.K. (2002). Profiles of airborne fungi in buildings and outdoor environments in the united states. Appl. Environ. Microbiol..

[47] O’Connor G.T., Walter M., Mitchell H., Kattan M., Morgan W.J., Gruchalla R.S., Pongracic J.A., Smartt E., Stout J.W., Evans R. (2004). Airborne fungi in the homes of children with asthma in low-income urban communities: The inner-city asthma study. J. Allergy Clin. Immunol..

[48] Zhang Y., Wang S., Li X.M., Cui C.M., Feng C., Wang B.G. (2007). New sphingolipids with a previously unreported 9-methyl-c20-sphingosine moiety from a marine algous endophytic fungus *Aspergillus niger* en-13. Lipids.

[49] Chaudhary V., Albacker L.A., Deng S., Chuang Y.T., Li Y., Umetsu D.T., Savage P.B. (2013). Synthesis of fungal glycolipid asperamide B and investigation of its ability to stimulate natural killer T cells. Org. Lett..

[50] Reibman J., Marmor M., Filner J., Fernandez-Beros M.E., Rogers L., Perez-Perez G.I., Blaser M.J. (2008). Asthma is inversely associated with helicobacter pylori status in an urban population. PLoS One.

[51] Chang Y.J., Kim H.Y., Albacker L.A., Lee H.H., Baumgarth N., Akira S., Savage P.B., Endo S., Yamamura T., Maaskant J. (2011). Influenza infection in suckling mice expands an NKT cell subset that protects against airway hyperreactivity. J. Clin. Invest..

[52] Stritesky G.L., Jameson S.C., Hogquist K.A. (2012). Selection of self-reactive T cells in the thymus. Annu. Rev. Immunol..

[53] Pei B., Speak A.O., Shepherd D., Butters T., Cerundolo V., Platt F.M., Kronenberg M. (2011). Diverse endogenous antigens for mouse NKT cells: Self-antigens that are not glycosphingolipids. J. Immunol..

[54] Zhou D., Mattner J., Cantu C., Schrantz N., Yin N., Gao Y., Sagiv Y., Hudspeth K., Wu Y.P., Yamashita T. (2004). Lysosomal glycosphingolipid recognition by NKT cells. Science.

[55] Christiansen D., Milland J., Mouhtouris E., Vaughan H., Pellicci D.G., McConville M.J., Godfrey D.I., Sandrin M.S. (2008). Humans lack iGb3 due to the absence of functional iGb3-synthase: Implications for NKT cell development and transplantation. PLoS Biol..

[56] Sanderson J.P., Brennan P.J., Mansour S., Matulis G., Patel O., Lissin N., Godfrey D.I., Kawahara K., Zahringer U., Rossjohn J. (2013). Cd1d protein structure determines species-selective antigenicity of isoglobotrihexosylceramide (iGb3) to invariant NKT cells. Eur. J. Immunol..

[57] Porubsky S., Speak A.O., Luckow B., Cerundolo V., Platt F.M., Grone H.J. (2007). Normal development and function of invariant natural killer T cells in mice with isoglobotrihexosylceramide (iGb3) deficiency. Proc. Natl. Acad. Sci. USA.

[58] Porubsky S., Speak A.O., Salio M., Jennemann R., Bonrouhi M., Zafarulla R., Singh Y., Dyson J., Luckow B., Lehuen A. (2012). Globosides but not isoglobosides can impact the development of invariant NKT cells and their interaction with dendritic cells. J. Immunol..

[59] Cox D., Fox L., Tian R.Y., Bardet W., Skaley M., Mojsilovic D., Gumperz J., Hildebrand W. (2009). Determination of cellular lipids bound to human CD1d molecules. PLoS One.

[60] Fox L.M., Cox D.G., Lockridge J.L., Wang X.H., Chen X.X., Scharf L., Trott D.L., Ndonye R.M., Veerapen N., Besra G.S. (2009). Recognition of lyso-phospholipids by human natural killer t lymphocytes. PLoS Biol..

[61] Chang D.H., Deng H., Matthews P., Krasovsky J., Ragupathi G., Spisek R., Mazumder A., Vesole D.H., Jagannath S., Dhodapkar M.V. (2008). Inflammation-associated lysophospholipids as ligands for CD1d-restricted T cells in human cancer. Blood.

[62] Lopez-Sagaseta J., Sibener L.V., Kung J.E., Gumperz J., Adams E.J. (2012). Lysophospholipid presentation by CD1d and recognition by a human natural killer t-cell receptor. EMBO J..

[63] Facciotti F., Ramanjaneyulu G.S., Lepore M., Sansano S., Cavallari M., Kistowska M., Forss-Petter S., Ni G.H., Colone A., Singhal A. (2012). Peroxisome-derived lipids are self antigens that stimulate invariant natural killer T cells in the thymus. Nat. Immunol..

[64] Brennan P.J., Tatituri R.V.V., Brigl M., Kim E.Y., Tuli A., Sanderson J.P., Gadola S.D., Hsu F.F., Besra G.S., Brenner M.B. (2011). Invariant natural killer T cells recognize lipid self antigen induced by microbial danger signals. Nat. Immunol..

[65] Stanic A.K., de Silva A.D., Park J.J., Sriram V., Ichikawa S., Hirabyashi Y., Hayakawa K., Van Kaer L., Brutkiewicz R.R., Joyce S. (2003). Defective presentation of the CD1d1-restricted natural va14ja18 NKT lymphocyte antigen caused by beta-d-glucosylceramide synthase deficiency. Proc. Natl. Acad. Sci. USA.

[66] Miyamoto K., Miyake S., Yamamura T. (2001). A synthetic glycolipid prevents autoimmune encephalomyelitis by inducing T(h)2 bias of natural killer T cells. Nature.

[67] Yu K.O.A., Im J.S., Molano A., Dutronc Y., Illarionov P.A., Forestier C., Fujiwara N., Arias I., Miyake S., Yamamura T. (2005). Modulation of CD1d-restricted NKT cell responses by using n-acyl variants of alpha-galactosylceramides. Proc. Natl. Acad. Sci. USA.

[68] Forestier C., Takaki T., Molano A., Im J.S., Baine I., Jerud E.S., Illarionov P., Ndonye R., Howell A.R., Santamaria P. (2007). Improved outcomes in nod mice treated with a novel th2 cytokine-biasing NKT cell activator. J. Immunol..

[69] Velmourougane G., Raju R., Bricard G., Im J.S., Besra G.S., Porcelli S.A., Howell A.R. (2009). Synthesis and evaluation of an acyl-chain unsaturated analog of the th2 biasing, immunostimulatory glycolipid, och. Bioorg. Med. Chem. Lett..

[70] Goff R.D., Gao Y., Mattner J., Zhou D.P., Yin N., Cantu C., Teyton L., Bendelac A., Savage P.B. (2004). Effects of lipid chain lengths in alpha-galactosylceramides on cytokine release by natural killer T cells. J. Am. Chem. Soc..

[71] Oki S., Chiba A., Yamamura T., Miyake S. (2004). The clinical implication and molecular mechanism of preferential IL-4 production by modified glycolipid-stimulated NKT cells. J. Clin. Inv..

[72] Fujio M., Wu D.G., Garcia-Navarro R., Ho D.D., Tsuji M., Wong C.H. (2006). Structure-based discovery of glycolipids for CD1d-mediated NKT cell activation: Tuning the adjuvant *versus* immunosuppression activity. J. Am. Chem Soc..

[73] Chang Y.J., Huang J.R., Tsai Y.C., Hung J.T., Wu D., Fujio M., Wong C.H., Yu A.L. (2007). Potent immune-modulating and anticancer effects of NKT cell stimulatory glycolipids. Proc. Natl. Acad. Sci. USA.

[74] Schiefner A., Fujio M., Wu D., Wong C.H., Wilson I.A. (2009). Structural evaluation of potent NKT cell agonists: Implications for design of novel stimulatory ligands. J. Mol. Biol..

[75] Li X.M., Fujio M., Imamura M., Wu D., Vasan S., Wong C.H., Ho D.D., Tsuji M. (2010). Design of a potent CD1d-binding NKT cell ligand as a vaccine adjuvant. Proc. Natl. Acad. Sci. USA.

[76] Michieletti M., Bracci A., Compostella F., de Libero G., Mori L., Fallarini S., Lombardi G., Panza L. (2008). Synthesis of alpha-galactosyl ceramide (krn7000) and analogues thereof via a common precursor and their preliminary biological assessment. J. Org. Chem..

[77] Toba T., Murata K., Futamura J., Nakanishi K., Takahashi B., Takemoto N., Tomino M., Nakatsuka T., Imajo S., Goto M. (2012). Synthesis and biological evaluation of truncated alpha-galactosylceramide derivatives focusing on cytokine induction profile. Bioorg. Med. Chem..

[78] Leung L., Tomassi C., Van Beneden K., Decruy T., Trappeniers M., Elewaut D., Gao Y., Elliott T., Al-Shamkhani A., Ottensmeier C. (2009). The synthesis and *in vivo* evaluation of 2 ',2'-difluoro krn7000. ChemMedChem.

[79] Bricard G., Venkataswamy M.M., Yu K.O.A., Im J.S., Ndonye R.M., Howell A.R., Veerapen N., Illarionov P.A., Besra G.S., Li Q.A. (2010). Alpha-galactosylceramide analogs with weak agonist activity for human iNKT cells define new candidate anti-inflammatory agents. PLoS One.

[80] Lee Y.S., Lee K.A., Lee J.Y., Kang M.H., Song Y.C., Baek D.J., Kim S., Kang C.Y. (2011). An alpha-galcer analogue with branched acyl chain enhances protective immune responses in a nasal influenza vaccine. Vaccine.

[81] Borg N.A., Wun K.S., Kjer-Nielsen L., Wilce M.C.J., Pellicci D.G., Koh R., Besra G.S., Bharadwaj M., Godfrey D.I., McCluskey J. (2007). Cd1d-lipid-antigen recognition by the semi-invariant NKT t-cell receptor. Nature.

[82] Trappeniers M., Goormans S., van Beneden K., Decruy T., Linclau B., Al-Shamkhani A., Elliott T., Ottensmeier C., Werner J.M., Elewaut D. (2008). Synthesis and *in vitro* evaluation of alpha-galcer epimers. ChemMedChem.

[83] Ndonye R.M., Izmirian D.P., Dunn M.F., Yu K.O., Porcelli S.A., Khurana A., Kronenberg M., Richardson S.K., Howell A.R. (2005). Synthesis and evaluation of sphinganine analogues of KRN7000 and OCH. J. Org. Chem..

[84] Leung L., Tomassi C., van Beneden K., Decruy T., Elewaut D., Elliott T., Al-Shamkhani A., Ottensmeier C., van Calenbergh S., Werner J. (2008). Synthesis and *in vivo* evaluation of 4-deoxy-4,4-difluoro-krn7000. Org. Lett..

[85] Park J.J., Lee J.H., Ghosh S.C., Bricard G., Venkataswamy M.M., Porcelli S.A., Chung S.K. (2008). Synthesis of all stereoisomers of krn7000, the CD1d-binding NKT cell ligand. Bioorg. Med. Chem. Lett..

[86] Dangerfield E.M., Cheng J.M.H., Knight D.A., Weinkove R., Dunbar P.R., Hermans I.F., Timmer M.S.M., Stocker B.L. (2012). Species-specific activity of glycolipid ligands for invariant NKT cells. Chembiochem.

[87] Hunault J., Diswall M., Frison J.C., Blot V., Rocher J., Marionneau-Lambot S., Oullier T., Douillard J.Y., Guillarme S., Saluzzo C. (2012). 3-fluoro- and 3,3-difluoro-3,4-dideoxy-krn7000 analogues as new potent immunostimulator agents: Total synthesis and biological evaluation in human invariant natural killer T cells and mice. J. Med. Chem..

[88] Lee T., Cho M., Ko S.Y., Youn H.J., Baek D.J., Cho W.J., Kang C.Y., Kim S. (2007). Synthesis and evaluation of 1,2,3-triazole containing analogues of the immunostimulant alpha-galcer. J. Med. Chem..

[89] Shiozaki M., Tashiro T., Koshino H., Nakagawa R., Inoue S., Shigeura T., Watarai H., Taniguchi M., Mori K. (2010). Synthesis and biological activity of ester and ether analogues of alpha-galactosylceramide (KRN7000). Carbohydr. Res..

[90] Sun M., Wang Y.H., Ye X.S. (2013). Design and synthesis of new krn7000 analogues. Tetrahedron.

[91] Tashiro T., Hongo N., Nakagawa R., Seino K., Watarai H., Ishii Y., Taniguchi M., Mori K. (2008). Rcai-17, 22, 24–26, 29, 31, 34–36, 38–40, and 88, the analogs of krn7000 with a sulfonamide linkage: Their synthesis and bioactivity for mouse natural killer T cells to produce th2-biased cytokines. Bioorg. Med. Chem..

[92] Fan G.T., Pan Y.S., Lu K.C., Cheng Y.P., Lin W.C., Lin S., Lin C.H., Wong C.H., Fang J.M., Lin C.C. (2005). Synthesis of alpha-galactosyl ceramide and the related glycolipids for evaluation of their activities on mouse splenocytes. Tetrahedron.

[93] Schmieg J., Yang G.L., Franck R.W., Tsuji M. (2003). Superior protection against malaria and melanoma metastases by a C-glycoside analogue of the natural killer T cell ligand alpha-galactosylceramide. J. Exp. Med..

[94] Tashiro T. (2012). Structure-activity relationship studies of novel glycosphingolipids that stimulate natural killer t-cells. Biosci. Biotech. Bioch..

[95] Kopecky-Bromberg S.A., Fraser K.A., Pica N., Carnero E., Moran T.M., Franck R.W., Tsuji M., Palese P. (2009). Alpha-C-galactosylceramide as an adjuvant for a live attenuated influenza virus vaccine. Vaccine.

[96] Zajonc D.M., Cantu C., Mattner J., Zhou D.P., Savage P.B., Bendelac A., Wilson I.A., Teyton L. (2005). Structure and function of a potent agonist for the semi-invariant natural killer T cell receptor. Nat. Immunol..

[97] Chen G., Chien M., Tsuji M., Franck R.W. (2006). E and Z alpha-C-galactosylceramides by julia-lythgoe-kocienski chemistry: A test of the receptor-binding model for glycolipid immunostimulants. Chembiochem.

[98] Li X.M., Chen G.W., Garcia-Navarro R., Franck R.W., Tsuji M. (2009). Identification of C-glycoside analogues that display a potent biological activity against murine and human invariant natural killer T cells. Immunology.

[99] Sullivan B.A., Nagarajan N.A., Wingender G., Wang J., Scott I., Tsuji M., Franck R.W., Porcelli S.A., Zajonc D.M., Kronenberg M. (2010). Mechanisms for glycolipid antigen-driven cytokine polarization by valpha14i NKT cells. J. Immunol..

[100] Patel O., Cameron G., Pellicci D.G., Liu Z., Byun H.S., Beddoe T., McCluskey J., Franck R.W., Castano A.R., Harrak Y. (2011). NKT cell recognition of CD1d-alpha-C-galactosylceramide. J. Immunol..

[101] Blauvelt M.L., Khalili M., Jaung W., Paulsen J., Anderson A.C., Brian Wilson S., Howell A.R. (2008). Alpha-s-galcer: Synthesis and evaluation for iNKT cell stimulation. Bioorg. Med. Chem. Lett..

[102] Dere R.T., Zhu X. (2008). The first synthesis of a thioglycoside analogue of the immunostimulant krn7000. Org. Lett..

[103] Hogan A.E., O’Reilly V., Dunne M.R., Dere R.T., Zeng S.G., O’Brien C., Amu S., Fallon P.G., Exley M.A., O’Farrelly C. (2011). Activation of human invariant natural killer T cells with a thioglycoside analogue of alpha-galactosylceramide. Clin. Immunol..

[104] Xing G.W., Wu D., Poles M.A., Horowitz A., Tsuji M., Ho D.D., Wong C.H. (2005). Synthesis and human NKT cell stimulating properties of 3-o-sulfo-alpha/beta-galactosylceramides. Bioorg. Med. Chem..

[105] Barbieri L., Costantino V., Fattorusso E., Mangoni A., Aru E., Parapini S., Taramelli D. (2004). Immunomodulatory α-galactoglycosphingolipids: Synthesis of a 2'-o-methyl-α-gal-gsl and evaluation of its immunostimulating capacity. Eur. J. Org. Chem..

[106] Veerapen N., Leadbetter E.A., Brenner M.B., Cox L.R., Besra G.S. (2010). Synthesis of a novel alpha-galactosyl ceramide haptenated-lipid antigen, a useful tool in demonstrating the involvement of iNKT cells in the production of antilipid antibodies. Bioconjugate Chem..

[107] Wu D., Xing G.W., Poles M.A., Horowitz A., Kinjo Y., Sullivan B., Bodmer-Narkevitch V., Plettenburg O., Kronenberg M., Tsuji M. (2005). Bacterial glycolipids and analogs as antigens for CD1d-restricted NKT cells. Proc. Natl. Acad. Sci. USA.

[108] Raju R., Castillo B.F., Richardson S.K., Thakur M., Severins R., Kronenberg M., Howell A.R. (2009). Synthesis and evaluation of 3''- and 4''-deoxy and -fluoro analogs of the immunostimulatory glycolipid, krn7000. Bioorg. Med. Chem. Lett..

[109] Xia C., Zhang W., Zhang Y., Chen W., Nadas J., Severin R., Woodward R., Wang B., Wang X., Kronenberg M. (2009). The roles of 3' and 4' hydroxy groups in alpha-galactosylceramide stimulation of invariant natural killer T cells. ChemMedChem.

[110] Zhang W., Xia C., Nadas J., Chen W., Gu L., Wang P.G. (2011). Introduction of aromatic group on 4'-oh of alpha-galcer manipulated NKT cell cytokine production. Bioorg. Med. Chem..

[111] Silk J.D., Salio M., Reddy B.G., Shepherd D., Gileadi U., Brown J., Masri S.H., Polzella P., Ritter G., Besra G.S. (2008). Cutting edge: Nonglycosidic CD1d lipid ligands activate human and murine invariant NKT cells. J. Immunol..

[112] Prigozy T.I., Naidenko O., Qasba P., Elewaut D., Brossay L., Khurana A., Natori T., Koezuka Y., Kulkarni A., Kronenberg M. (2001). Glycolipid antigen processing for presentation by CD1d molecules. Science.

[113] Zhou X.T., Forestier C., Goff R.D., Li C., Teyton L., Bendelac A., Savage P.B. (2002). Synthesis and NKT cell stimulating properties of fluorophore- and biotin-appended 6"-amino-6"-deoxy-galactosylceramides. Org. Lett..

[114] Xia C., Zhang W., Zhang Y., Woodward R.L., Wang J., Wang P.G. (2009). Facile synthesis of biotin-labelled α-galactosylceramide as antigen for invariant natural killer T cells. Tetrahedron.

[115] Cheng J.M., Chee S.H., Knight D.A., Acha-Orbea H., Hermans I.F., Timmer M.S., Stocker B.L. (2011). An improved synthesis of dansylated alpha-galactosylceramide and its use as a fluorescent probe for the monitoring of glycolipid uptake by cells. Carbohydr. Res..

[116] Liu Y., Deng S., Bai L., Freigang S., Mattner J., Teyton L., Bendelac A., Savage P.B. (2008). Synthesis of diglycosylceramides and evaluation of their iNKT cell stimulatory properties. Bioorg. Med. Chem. Lett..

[117] Liu Y., Goff R.D., Zhou D., Mattner J., Sullivan B.A., Khurana A., Cantu C., Ravkov E.V., Ibegbu C.C., Altman J.D. (2006). A modified alpha-galactosyl ceramide for staining and stimulating natural killer T cells. J. Immunol. Methods.

[118] Trappeniers M., van Beneden K., Decruy T., Hillaert U., Linclau B., Elewaut D., van Calenbergh S. (2008). 6'-derivatised alpha-galcer analogues capable of inducing strong CD1d-mediated th1-biased NKT cell responses in mice. J. Am. Chem. Soc..

[119] Aspeslagh S., Li Y., Yu E.D., Pauwels N., Trappeniers M., Girardi E., Decruy T., van Beneden K., Venken K., Drennan M. (2011). Galactose-modified iNKT cell agonists stabilized by an induced fit of CD1d prevent tumour metastasis. EMBO J..

[120] Jervis P.J., Graham L.M., Foster E.L., Cox L.R., Porcelli S.A., Besra G.S. (2012). New CD1d agonists: Synthesis and biological activity of 6''-triazole-substituted alpha-galactosyl ceramides. Bioorg. Med. Chem. Lett..

[121] Jervis P.J., Moulis M., Jukes J.P., Ghadbane H., Cox L.R., Cerundolo V., Besra G.S. (2012). Towards multivalent CD1d ligands: Synthesis and biological activity of homodimeric alpha-galactosyl ceramide analogues. Carbohydr. Res..

[122] Tashiro T., Nakagawa R., Hirokawa T., Inoue S., Watarai H., Taniguchi M., Mori K. (2007). Rcai-56, a carbocyclic analogue of krn7000: Its synthesis and potent activity for natural killer (nk) T cells to preferentially produce interferon-γ. Tetrahedron Lett..

[123] Tashiro T., Sekine-Kondo E., Shigeura T., Nakagawa R., Inoue S., Omori-Miyake M., Chiba T., Hongo N., Fujii S., Shimizu K. (2010). Induction of th1-biased cytokine production by alpha-carba-galcer, a neoglycolipid ligand for NKT cells. Int. Immunol..

[124] Yoshiga Y., Goto D., Segawa S., Horikoshi M., Hayashi T., Matsumoto I., Ito S., Taniguchi M., Sumida T. (2011). Activation of natural killer T cells by alpha-carba-galcer (rcai-56), a novel synthetic glycolipid ligand, suppresses murine collagen-induced arthritis. Clin. Exp. Immunol..

[125] Harrak Y., Barra C.M., Bedia C., Delgado A., Castano A.R., Llebaria A. (2009). Aminocyclitol-substituted phytoceramides and their effects on iNKT cell stimulation. ChemMedChem.

[126] Kerzerho J., Yu E.D., Barra C.M., Alari-Pahissa E., Girardi E., Harrak Y., Lauzurica P., Llebaria A., Zajonc D.M., Akbari O. (2012). Structural and functional characterization of a novel nonglycosidic type i NKT agonist with immunomodulatory properties. J. Immunol..

